# An ethnoveterinary study of medicinal plants in treatment of diseases and syndromes of herd dog in southern regions of Ilam province, Iran

**DOI:** 10.1007/s00580-012-1423-8

**Published:** 2012-01-26

**Authors:** Mahmoud Bahmani, Zohre Eftekhari

**Affiliations:** 1 Young Researchers Club, Dehloran Branch, Islamic Azad University, Dehloran, Iran; 2 Veterinary Medicine Faculty, Tehran University, Tehran, Iran

**Keywords:** Medicinal plants, Southern area of Ilam province, Herd dog

## Abstract

This paper describes a selection of the ethnoveterinary medicines used for herd dogs in the southern regions of Ilam province, Iran. Traditional botanical medicine is the primary mode of healthcare for most of the rural population in Ilam province. In this study, a questionnaire was distributed among 45 residential areas in 22 rural zones of the southern areas of Ilam province. The objective of this study was the recognition of natural medicinal methods using medicinal plants, and the classification of ethnoveterinary applications and collection of domestic science. Twenty-two medicinal plants from 16 families were identified. The main application of these plants was for the detection and treatment of digestive disorders using *Citrullus colocynthis*, *Aristolochia clematis*, *Scrophularia deserti*, *Quercus brantii*, *Ceracus microcarpa*, *Echium strigosa*, *Pistacia atlantica*, and *Pistacia khinjuk* which have been applied using *Euphurbia graminifolius*, *Peganum harmala*, *Salsola rigida*, *Artemisia herba-alba*, *Amygdalus arabica*, jolbak of salt water, *Peganum harmala* L., and *Nicotina tabacum* for external and internal parasite disorders. *S. deserti* for ophthalmic disorders, and *P. atlantica*, *P. khinjuk*, and *Q. brantii* for respiratory disorders were applied. The present study confirmed the traditional medical effects of some plants and revealed the unique medical effects of other plants, which if recognized could be useful in the creation of new ideas and increasing knowledge for the modern pharmaceutical industry. Since very few clinical trials have been conducted on plants native to Ilam province, it is necessary that more research be conducted to ensure that labeled and standardized products are introduced for human consumption.

## Introduction

According to ancient medical and veterinary literature, native information of herdsmen regarding the usage of medicinal plants in animal and human disorders is described as ethnoveterinary medicine (EVM). EVM considers that traditional practices of veterinary medicine are legitimate and seeks to validate them. Sources of medicinal plants and pharmaceuticals in many countries such as Iran, China, ancient Greece, India, and Egypt have long been used in the treatment and diagnosis of different diseases (Ghasemi Pirbalouti 2009; Ghasemi Pirbalouti et al. 2009).

According to the World Health Organization (WHO), plants are a supplier of medicines for human consumption (Ghasemi Pirbalouti 2009; Ghasemi Pirbalouti et al. 2009; WHO 2001). Factors such as lack of access to the majority of chemical drugs and/or the cost of chemical drugs, as well as the adverse side effects of chemical drugs, lead to the advantages of using herbal drugs instead of chemical medicines (Ghasemi Pirbalouti 2009; Ghasemi Pirbalouti et al. 2009). Herbal remedies used for hundreds of years by stock raisers could be put to commercial use, but scientists are demanding that traditional knowledge be validated, to verify the safety and efficacy of the treatments. This study was carried out to identify traditional plants used in the treatment of disorders based on Iran’s rich history in traditional and herbal medicine derived from the treatments of the famous Iranian scientists such as Avicenna, as well as the various customs of the different ethnic groups in Iran.

Southern regions of Ilam are part of a common market in pharmaceuticals and are involved in both the major cattle and sheep farmlands, and herd animals are often in transition from other regions. One of the main goals of this research was to investigate commonalities in ethnoveterinary medicine in these regions and to introduce new herbal drugs that can be appropriate in the treatment of disorders in human and domestic animals.

## Material and methods

### Study area: south area of Ilam province

Ilam is located in the west of Iran at latitude of 33°38′ north and longitude of 46°26′ east, in a cold, mountainous region 1,319 m above mean sea level. Although this city is surrounded by mountains, its climate is also affected by deserts from the west and the south. This region presents a highly variable annual weather profile. Heavy showers or heavy snow in the winter, and dusty, hot, dry weather in the summer are quite normal.

Abdanan is a county in Ilam province, Iran. At the time of the 2006 census, its population was 45,830 in 9,358 families. Dehloran is another county in Ilam Province, Iran. At the 2006 census, the population in this county was 58,993 in 11,376 families.

### Data collection

Data collection and analysis of plants took place in the southern regions of Ilam province from 2009 to 2010. This paper describes a selection of the ethnoveterinary medicines that were used for herd dogs in these regions. Herd dogs, used for protecting livestock, are quite important, particularly among traditional farmers. Very little research has been conducted on ethnoveterinary medicine being used for dogs, and there are few comparative studies. The respondents were ethnically and demographically varied. In support of this purpose, the questionnaires for the five groups of diseases and syndromes such as gastrointestinal, cutaneous, parasitic, respiratory, and nervous disorders of sheepdog were designed. The questionnaires were distributed among 45 persons (7 men and 38 women) of traditional farmers from 22 villages in the southern province of Ilam with an age range of 55–75 years. The questionnaire contents included information such as local name, plant parts used, traditional usage, and traditional therapeutic effects. In addition to achieving information regarding the plants being used for treatment and about the area, a sample of the herb that was used was collected.

The plant-based remedies were evaluated for safety and efficacy with a nonexperimental method, prior to including them in the draft outline. Published sources such as journal articles and books, and databases on pharmacology, ethnopharmacology, ethnobotany, and ethnomedicine available on the Internet were searched to identify the plants’ chemical compounds and clinically tested physiological effects. The ethnoveterinary usages of locally available plants for herd dog in Ilam province are summarized in Table 1.

**Table 1:** Ethenoveterinary medicines used for dogs in southern regions of Ilam province

Scientific name	Family	Plant part used	Native name	Persian name	Use (Ghasemi Pirbalouti 2009)
*P. harmala* L.	Zygophyllaceae	Seed	Esphan	Esfand	Snake biting
*E. graminifolius*	Compositae	Soden	Shirkosh	Sheng	Intestinal worm
*S. rigida*	Solanaceae	Aerial part	Shoor	Alafe shoor	Intestinal worm
*A. herba-alba*	Compositae	Aerial part	Bookhoshkele	Dermaneh	Intestinal worm
*A. vera*	Liliaceae	Leaf	Sabre zard	Sabre zard	Laxative
*Aristolochia clematis*	Aristolochiaceae	Aerial part	Zaravand	Zaravand	Washing the ulcer
*C. album* L.	Cheopodiaceae	Aerial part	Solme tare	Solme tare	Laxative
*D. orientalis* (Gay) Schrod.	Ranunculaceae	Aerial part	Zaban dar ghafa	Zaban dar ghafaye sharghi	Laxative
*S. deserti* Del.	Scrophulariaceae	Aerial part	Benjek mashine	Gole meymoni biabani	Disinfection of wounds
*A. arabica* Olivier	Rosaceae	Fruit	Bayem	Badam bibarg	Tick, lice, intestinal worms
*Q. brantii* Lindl. var. *persica* (Jaub & Spach) Zohary	Fagaceae	Fruit	Bali	Balout irani	Diarrhea, coughing, mouth ulcer
*E. strigosa* Labill.	Boraginaceae	Flower	Gole gazo	Gavzaban kharakdar	Abscess
Jolbak of salt water	Coralinaceae	Total part	Gazaf	Jolbak	Snake biting
*P. atlantica* Desf and *P. khinjuk* Stocks	Anacardiaceae	Areal part	Bonak	Bane	Coughing
*S. khuzistanica* Jamzed	Labiatae	Stem and leaf	Jatare	Marzeye khozestani	Antileech
*N. tabacum*	Solanaceae	Aerial part	Tamako	Tanbacco	Antileech
*Olivera decumbens* Vent.	Umbelliferae	Aerial part	None khoda	Laele kohestan	Diarrhea
*S. luteum*	Solanaceae	Fruit	Ra’razek	Tajrizi	Diarrhea
*Citrus limonum*	–	Fruit	Limo	Limo	Diarrhea
*Astakhys lavandili* folia Vahl	Labiatae	Aerial part	Kolpar	Chaye kohi	Diarrhea
*Ceracus microcarpa* (C. A. Mey.) Boiss.	Rosaseae	Leaf	Malho	Mahlab	Antiseptic and disinfection of wounds
*C. colocynthis* (L.) Schard	Cucubitaceae	Fruit and flower	Gijalak	Hendavane abojahl	Antiseptic and wound healing

## Results and discussion

The importance of gender is being increasingly recognized in EVM. One of the first studies to document gender was conducted by Diana Davis, who noted a difference in knowledge of EVM of Afghan Pashtun nomads that paralleled the gender-based division in the society. Davis found that women know more about healthcare for newborns and very sick animals that are taken care of near the home (Davis 2000). Since women prepare the carcass for consumption, they know twice as many types of internal parasites as men. Women also help with dystocias and the manual removal of ectoparasites. In the present study, most of the information pertaining to the usages of herbal drugs was given by women, who are more knowledgeable about healthcare for sick animals that are taken care of near the home.

In a research conducted in Trinidad, it was noted that male farmers were using the reproductive knowledge of their female relatives to assist in the health care of their ruminants. Female farmers were using the same plants for their animals that they used for themselves (Lans et al. 2006).

It was found that in total, 22 plants were being used in the treatment of dog disorders (Table 1). Some of the plants in this study were poisonous. These are shown in Table 2. Recognizing the components of each plant in all regions of a country can aid in our understanding of renewable natural resources and contribute effectively, in line with the characteristics and uses of medicinal plants and their uses (Ghasemi Pirbalouti 2009; Ghasemi Pirbalouti et al. 2009). In the present study, some plants which are used for dog disorders such as *Delphinium orientalis* (Gay) Schrod, *Artemisia herba-alba*, *Nicotina tabacum*, *Echium strigosa* Labill, *Saturiya khuzistanica* Jamzed, *Aloe vera*, *Scrophularia deserti* Del, and *Citrullus colocynthis* (L.) Schard have some new effects which have not previously been mentioned, but other plants which are used in the treatment of dog disorders such as *A. vera* and *E. strigosa* in new herbal medicine are mentioned (Amouzgar et al. 2008; Jahanara and Haerizadeh 2001; Rojhan 2001; Samsam Shariat and Moatar 1975; Zargari 1994).

**Table 2:** Additional information about poisonous plants

Scientific name	Family	Toxic component (Ghasemi Pirbalouti 2009)
*P. harmala*	Zygophyllaceae	Norharman, harman, …
*A. arabica*	Rosaceae	Amygdalin
*Q. brantii*	Fagaceae	Gallotannin
*A. herba-alba*	Astraceae	Artemisinin
*C. album*	Cheopodiaceae	Oxalate, cyanogenic glycosides and nitrate
*S. rigida*	Solanaceae	Oxalate
*Echium* spp.	Boraginaceae	Pyrrolizidine alkaloids
*S. luteum*	Solanaceae	Solanine
*N. tabacum*	Solanaceae	Nicotine

*Euphurbia graminifolius*, *Salsola rigida*, *Amygdalus arabica* Olivier, and *A. herba-alba* have been widely used for elimination of intestinal worms, and in most areas, these plants are used for the same purpose in other animals as well. *A. vera*, *Chenopodium album* L., *D. orientalis* (Gay) Schrod were used as a laxative in constipation, and can be lucrative for treatment of accumulation of rumen, reticulum, and omasum in ruminant rather than chemical drugs. The use of *Pistacia atlantica* Desf, *Pistacia khinjuk* Stocks, and *Quercus brantii* Lindl. var. *persica* (Jaub & Spach) Zohary by farmers for the treatment and alleviation of coughing in dogs can be useful in the introduction of a new generation of herbal drugs for human and domestic animals.

*A. herba-alba* is good fodder for grazing animals, mainly sheep and cattle. This species of sagebrush is widely used in folk and traditional medicine for its antiseptic and antispasmodic properties.

*A. herba-alba* is reported as a traditional remedy of enteritis, and various intestinal disturbances. The essential oil from *Satureja khuzestanica* Jamzad (SKEO), an endemic plant from Iran, was evaluated for its activity against inflammatory bowel disease (IBD). SKEO was examined on the experimental mouse model of IBD, which is acetic acid-induced colitis.

*Peganum harmala* is used as an analgesic and anti-inflammatory agent, as well as in the treatment of depression. It has antibacterial activity against drug-resistant bacteria and is used in the treatment of syphilis in India, fever in North Africa, hysteria, neuralgia, Parkinson’s disease, prolapsed uterus, rheumatism, asthma, and eye irritation. *P. harmala* is an abortifacient and is effective against protozoa including malaria (Al-Sharma et al. 1981; Ahmad et al. 1992; El-Rifaie 1980).

Zargari reported that *E. graminifolius* is used to treat dysentery. Its extracts in healing stomach ulcers and liver disorders are useful. Additionally, Zachariah recommended this plant for disposal of pesticides (Zargari 1994). Scientific evidence for the cosmetic and therapeutic effectiveness of *A. vera* is limited. *A. vera* juice is used for consumption and relief of digestive issues such as heartburn and irritable bowel syndrome, although it bears significant potential to be toxic when taken orally. Other uses for extracts of *A. vera* include the dilution of semen for the artificial fertilization of sheep, use as a fresh food preservative, and is used for water conservation in small farms. Therapeutic effects of *A. vera* include treatment of arthritis, asthma, candidiasis, treatment of chronic fatigue, indigestion and intestinal disorders (colitis and ulcerative colitis), skin disorders (psoriasis, acne, burns, infections, foot fungus, skin damage caused by cold), sports injuries, and wounds. Internal and external uses of *A. vera* have a long association with herbal medicine, although it is not known when its medical applications were first suspected. *A. vera* may be effective in treatment of wounds. Some studies have shown that *A. vera* promotes the rates of healing. In addition to topical use in wound or burn healing, internal intake of *A. vera* has been linked in preliminary research with improved blood glucose levels in diabetics and with lower blood lipids in hyperlipidemic patients, as well as with acute hepatitis (liver disease). In other diseases, preliminary studies have suggested oral *A. vera* gel may reduce symptoms and inflammation in patients with ulcerative colitis. In the present study, internal intake of *A. vera* in dogs caused reduced constipation and is introduced as a laxative.

Compounds extracted from *A. vera* have been used as an immune stimulant that aids in fighting cancers in cats and dogs; however, this treatment has not been scientifically tested in humans. Topical application of *A. vera* may be effective for genital herpes and psoriasis. However, it is not effective for the prevention of radiation-induced injuries. And although anecdotally useful, it has not been proven to offer protection from sunburn.

*A. vera* extracts might have antibacterial and antifungal activities, which could possibly help treat minor skin infections such as boils and benign skin cysts, and may inhibit growth of fungi causing tinea. Inner leaf gel from *A. vera* was shown to inhibit the growth of *Streptococcus* and *Shigella* species in vitro (Chow et al. 2005).

*C. colocynthis* (L.) Schard has been linked in preliminary research with improved blood glucose levels in diabetics and digestive disorders, and the laxative effects of this plant have been proven (Asfi 1994; Tavakol Afshari et al. 2005). In Iranian traditional medicine, *lemon* was used for alleviating headache (Naseri et al. 2008), dizziness, and stomach inflammation, while almond was used to relieve acute urination and disorders of saliva.

Other usages of medical plants in ancient and traditional medicine included antimastitis effect of *harmal*, treatment of constipation, chronic wound healing, and treatment of infections of *P. atlantica* Desf and *P. khinjuk* Stocks. *Scrophularia scopolii* was used for relief of pulmonary disorders, gangrenous wounds, and back pain, but in the present study, *S. deserti* Del was used to disinfect wounds in skin and eyes (Ardakani Yazdi 2006).

In a study by Bahmani et al. (2011a, b), 35 plants were recognized for treatment of small ruminants’ disorders. In this study, the *peanut* plant was used for treatment of accumulation of rumen, *Q. brantii* Lindl. var. *persica* Zohary for treatment of diarrhea, cough, and mouth ulcers; *D. orientalis* (Gay) Schrod, Coralinaceae, and *A. vera* as a laxative (Jeremy et al. 2005; Ki et al.^1999^); *P. atlantica* Desf for treatment of *Oestrus ovis* larva; and *N. tabacum* as an antileech drug. *E. strigosa* Labill and *Q. brantii* Lindl. var. *persica* (Jaub & Spach) Zohary was used for treatment of mastitis (Bahmani et al. 2011a, b).

Bahmani et al. (2011a, b) and Ghasemi Pirbalouti (2009) demonstrated the anticandidiasis effect of *S. deserti* and *Scrophularia striata*. In another study, Bahmani et al. (2010, 2011a, b) revealed the antileech effects of the *tobacco* plant.

The present study confirmed the traditional medical effects of some plants, while revealing the unique medical effects of other plants .Recognizing such plants and their effects can aid in the creation of new ideas for increasing knowledge in the modern pharmaceutical industry. Since very few clinical trials have been conducted on plants that are native to Ilam province, it is vital that more research be done to ensure the introduction of labeled and standardized products for human consumption.

This ethnobotanical survey results revealed the wealth of indigenous knowledge and usage customs of traditional plants associated with the rural people of the southern regions of Ilam. Despite their use in traditional medicines, plant species renowned in the present fieldwork have been extensively used for improving the health of sheepdogs. There was no written certificate of traditional healing knowledge, and the transfer of such knowledge to the future generation takes place only through oral communication. More detailed ethnopharmacological investigations need to be conducted in this area, particularly concerning conservation strategies and sustainable use of medicinal plants.
